# Depressed Mood after Stroke: Predictive Factors at Six Months Follow-Up

**DOI:** 10.3390/ijerph17249542

**Published:** 2020-12-20

**Authors:** Fidel López-Espuela, Raúl Roncero-Martín, Maria de la Luz Canal-Macías, Jose M. Moran, Vicente Vera, Adela Gomez-Luque, Alejandro Lendinez-Mesa, Juan Diego Pedrera-Zamorano, Ignacio Casado-Naranjo, Jesus Lavado-García

**Affiliations:** 1 Metabolic Bone Diseases Research Group, Nursing Department, Nursing and Occupational Therapy College, University of Extremadura, 10003 Cáceres, Spain; fidellopez@unex.es (F.L.-E.); rronmar@unex.es (R.R.-M.); jmmorang@unex.es (J.M.M.); adelagl@unex.es (A.G.-L.); jpedrera@unex.es (J.D.P.-Z.); jmlavado@unex.es (J.L.-G.); 2 Department of Stomatology II, School of Dentistry, Complutense University, 28040 Madrid, Spain; viventevera@odon.ucm.es; 3 Nursing Department, Faculty of Health Sciences, Alfonso X el Sabio University, 28691 Madrid, Spain; alendmes@uax.es; 4 Department of Neurology, Complejo Hospitalario De Cáceres, 10004 Cáceres, Spain; icasadon@gmail.com

**Keywords:** cerebrovascular disease, depression, disability, Hamilton Depression Rating Scale, post-stroke depression, stroke

## Abstract

We aimed to know the prevalence of post-stroke depression (PSD) in our context, identify the variables that could predict post-stroke depression, by using the Hamilton Depression Rating Scale, occurring within six months after stroke, and identify patients at high risk for PSD. Methods: descriptive, cross-sectional and observational study. We included 173 patients with stroke (transient ischemic attack (TIA) included) and collected sociodemographic and clinical variables. We used the Hamilton Depression Scale (HDS) for depression assessment and Barthel Index and modified Rankin Scale (mRS) for functional assessment. The neurological severity was evaluated by the National Institutes of Health Stroke Scale (NIHSS). Results: 35.5% were women, aged 71.16 (±12.3). Depression was present in 42.2% patients (*n* = 73) at six months after stroke. The following variables were significantly associated with PSD: diagnosis of previous depression (*p* = 0.005), the modified Rankin Scale at discharge (*p* = 0.032) and length of hospital stay (*p* = 0.012). Conclusion: PSD is highly prevalent after stroke and is associated with the severity, left location of the stroke, and the degree of disability at discharge. Its impact justifies the evaluation and early treatment that still continues to be a challenge today.

## 1. Introduction

Stroke is the second largest cause of death worldwide and has the third largest impact on disability-adjusted life years [1]. The consequences of a stroke are diverse and can depend on factors such as type of stroke, affected brain area, age, comorbidities and time to treatment [2]. Subsequent disability may not only be physical but can also involve cognitive and psychological problems [1,3]. Thus, despite advancements in prevention and therapy, stroke remains both a serious human problem for patients and families and a dramatic public financial burden [4,5,6].

Post-stroke depression (PSD) is considered the most frequent and burdensome neuropsychiatric complication experienced following stroke. Meta-analyses have estimated the cross-sectional prevalence of PSD between 18 and 33% of stroke survivors [7,8] with a cumulative incidence of 55% [9]. However, it may happen that the number could be underrated due to exclusion, in the majority of studies, of patients with communication issues, aphasia or dementia. Persons with PSD have higher mortality rates [7,10], more pronounced cognitive deficits, more long-term disability, lower quality of life and higher rates of suicidal ideation as compared to post-stroke patients without depression, indicating that early detection and treating depression are important after a stroke [11,12,13,14]. Thus, the consequences of not detecting depression symptoms at an early stage can be relevant for the patient as well as health systems [12,15,16].

The diagnosis of PSD relies on five criteria: (a) presence of depressed mood or anhedonia; (b) symptoms are pathophysiologically related to the stroke; (c) symptoms are not better explained by other psychiatric disorders; (d) disturbance does not occur exclusively in the presence of delirium; (e) symptoms cause significant distress or impairment [17].

There are some notable differences between PSD and major depression (MD); the pathophysiology of PSD is closely linked to vascular injury [4]. PSD tends to have more severe depressive symptoms than MD does [18]. Main symptoms of PSD are depressive mood, more cognitive impairment, apathy, less anhedonia, inappetence, less sleep disturbance, decreased energy, self-blame, self-mutilation and even suicidal tendencies [4,18,19,20]. Individuals with PSD have a disproportionally high prevalence of physical disability such as aphasia, motor/gait impairment and sensory losses [13].

The etiology of PSD is poorly understood [21]. Some studies suggest a potentially bi-directional relationship between stroke and depression; stroke increases the risk of PSD, but depression is an independent risk factor for stroke and stroke mortality [4,22].

Neurobiological dysfunctions and psychosocial factors are considered as two major risk factors of PSD [4,23]. Studies have revealed an association between PSD and post-stroke cognitive and functional deficits, indirectly suggesting that PSD may be a psychological reaction to these deficits.

In addition, some research confirmed some pre-stroke risk factors for PSD such as female sex, personal history of psychiatric illness (particularly major depression and anxiety disorders), family history of psychiatric disorders, higher degree of neuroticism and social isolation [4,11,21]. The relationship between age and PSD still remains unclear [13]. New pre-stroke risk factors have also been suggested, including exposure to stressful life events prior to the stroke, diabetes mellitus, lower educational level and Latino ethnicity [24,25,26].

Evidence suggests that PSD has underlying biological causes and is not merely a psychological response to new disability or a life-threatening event. Proposed biological factors contributing to PSD include lesion location, genetic susceptibility, inflammation, excess of pro-inflammatory cytokines, neurogenesis in response to ischemia, alterations in neurotrophic factors, disruption of cortico-striato-pallido-thalamic-cortical projections as well as alterations in serotonergic, noradrenergic and dopaminergic pathways, leading to changes in amine levels [4,21,23].

This heterogeneity may result from the subtype of depression (major, minor), the post-stroke onset (early or late), the cerebral area involved (left versus right hemisphere) and the causal factors [27].

There are still some controversial issues in the PSD, so we want to expose what is going on in our environment. Therefore, PSD is significant not only due to its high prevalence but also its involvement and negative influence on the neurological, functional and cognitive recovery, as well as patients’ survival. Healthcare providers should evaluate depression and other psychological disorders during post-stroke follow-up visits.

The objective of the study was to know the prevalence of the post-stroke depression in our environment, identify the variables that could predict post-stroke depression, by means of using the Hamilton Depression Rating Scale, occurring within six months after stroke, and identify patients at high risk for PSD.

## 2. Materials and Methods

### 2.1. Design

This was a descriptive, cross-sectional and observational study, where patients surviving a stroke and consecutively attended the vascular neurology clinic of the Complejo Hospitalario in Cáceres were evaluated.

Patients were evaluated at the 6-month follow-up after their stroke in order to check their mood and state of health at that time. In addition, data related to hospital admission were collected (such as days of stay, assessment of the severity of the stroke, etc.).

### 2.2. Criteria for Inclusion

The inclusion criteria were patients with a definitive diagnosis of ischemic (TIA included) or hemorrhagic stroke, including patients with aphasia who were admitted to our hospital and who attended the 6-month follow-up session (all surviving patients attend this session). Those patients with cognitive impairment, previous dementia or history of psychosis were excluded.

### 2.3. Variables and Instruments

The assessment instruments used and the variables collected in the follow-up visit at 6 months were the following:

(a) Sociodemographic variables: age, gender, socioeconomic status, educational level and social assessment evaluated using the Gijon’s social-familial evaluation scale (SFES) [28]. The Gijon’s SFES assesses the subject’s household composition, net monthly household income, accommodation, social relationships and social support received. The Gijon’s SFES is a self-administered scale composed of five items, the maximum score being 25 points. Higher scores indicate a major social risk, scores between 10 and 14 indicate a social risk and scores over 15 social problems.

(b) Clinical variables: the neurologist reviewed the patient’s history prior to the stroke to find out their vascular risk factors and whether they were depressed prior to the stroke. In addition, information on the type of stroke was collected, whether fibrinolysis was performed (rtPA IV), location of the stroke and presence of hemiparesis at the hospital discharge. Information on stroke subtype (ischemic or hemorrhagic, based on clinical and neuroimaging findings) was also collected.

According to the Oxfordshire Community Stroke Project classification, acute ischemic stroke is divided into four subtypes: lacunar infarct, total anterior circulation infarct (TACI), partial anterior circulation infarct and posterior circulation infarct [29].

Comorbidity was assessed using the Charlson Comorbidity Index (CCI) [30,31]. The index contained 19 comorbid conditions and was adjusted specifically for stroke evaluation. A CCI score of 0–1 indicates low comorbidity, and a score of 2 or higher is considered high comorbidity, unless otherwise specified. Comorbid conditions were recorded if present in the medical record.

(c) Psychopathological assessment: we used the Hamilton Depression Scale (HDS) [32,33]. The scale in the Spanish version used in the study has 17 items, including depressed mood, feelings of guilt, attempted suicide, early insomnia, middle insomnia and late insomnia, work and activities, inhibition, agitation, psychic anxiety and somatic anxiety, gastrointestinal somatic symptoms, general somatic symptoms, sexual symptoms, hypochondria, weight loss and ability to understand.

The cutoff points to define the levels of severity of depression recommended by the American Psychiatric Association [34] are as follows: no depression (0–7 points); mild/minor depression (8–13 points); moderate depression (14–18 points); severe depression (19–22 points); very severe depression (more than 23 points). Each patient was screened at the six-month follow-up.

(d) Functional assessment: The ability to perform basic activities of daily life was evaluated by using the Barthel Index (BI) [35] consisting of 10 items. In the Barthel Index a total score between 0 and 20 suggests total dependence, between 21 and 60 severe dependence, between 61 and 90 moderate dependence, between 91 and 99 slight dependence and 100 independence.

The functional condition of the patient was collected by means of using the modified Rankin Scale (mRS) [36]; the mRS remains a popular, validated functional outcome measure in acute stroke trials and other stroke studies. Stroke outcome is most commonly rated by the mRS because of the validity and rapid application of this rating scale and its ability to discriminate clinically relevant levels of disability and recovery. It is an ordinal scale with seven categories: 0, no symptoms at all; scores >3 are defined as severe disability.

Data on these variables (BI and mRS) were collected from the patient’s medical history at discharge.

(e) Neurological assessment: The neurological severity was evaluated by the National Institutes of Health Stroke Scale (NIHSS). Scores were examined by experienced vascular neurologists at the time of admission [37]. NIHSS is a 15-item impairment scale for stroke severity measurement; the scoring range is 0 to 42 points, the higher the number, the greater the severity. A score of less than 5 represents no stroke symptoms or a minor stroke, a score of 5 to 15 represents a moderate stroke. This datum was collected in the medical history at admission.

### 2.4. Procedures

At the 6-month follow-up, patients (and/or proxies, in the case of patients with severe aphasia) were provided with the study information, and informed consent to participate was signed. The aforementioned questionnaires and variables were also completed. The patients were evaluated by a psychology graduate. The information was encrypted and decoupled to safeguard the confidentiality of the participants; this file was only accessible by the main researcher.

### 2.5. Ethics

The research was approved by the Ethical Committee of the area of Cáceres (REFCEIM1225), and all participants were previously informed. They understood the objectives and practices involved in the study, and they signed the written informed consent. The study was carried out in accordance with the ethical principles of the Declaration of Helsinki, and confidentiality of the data collected was guaranteed in accordance with current legislation.

### 2.6. Statistical Analysis

Quantitative variables were expressed by the mean and standard deviation or the median and interquartile range according to the normality of the distribution. Categorical variables were presented with their absolute and relative frequency. The association between qualitative variables was studied using the χ^2^ test and quantitative variables with the Mann–Whitney U test.

Multivariate analysis models were calculated by using linear logistic regression analysis. The variables that were evaluated and included in the univariate and multivariate logistic regression models to determine the predictive factors were as follows: age, gender, socioeconomic class, educational level, place of residence, social support (Gijon´s SFES), the Charlson comorbidity index, being previously diagnosed with depression, the type and side of the stroke, the score on the NIHSS at admission, presence of hemiparesis or hemiplegia, the score on the BI and on the mRS at discharge as well as length of hospital stay.

Data were analyzed by means of using SPSS v.20 (IBM Corp., Chicago, IL, USA). The statistical significance level was established at 0.05.

## 3. Results

During the study period, 173 patients were included. Demographic characteristics of the patients were as follows: 35.5% women, and the average age of the sample was 71.16 (±12.3) years. Regarding coexistence, 67.1% of patients lived with partners, 15% alone and the rest with family members. All participants were white.

Regarding cardiovascular risk factors, 61.8% had hypertension, 20.2% diabetes mellitus, 17.9% atrial fibrillation, 36.4% dyslipidemia, 26.5% heart disease and 25.4% had a previous stroke. At six months 53 patients (30.8%) had aphasia.

### Functional Status and Psychopathological Characteristics

At hospital discharge, patients had an average BI score of 76.42 (±29.19) and an mRS score of 2.20 (±1.53). At the 6-month follow up, the scores on these scales were 86.47 (±23.98) and 1.90 (±1.48), respectively, with significant differences (*p* < 0.001 in both cases).

Of the sample, 67.6% had a favorable functional prognosis (mRS < 3), and 86.1% scored above 60 points in the BI at 6 months, compared to 71.1% who had it at discharge.

Of the sample, 42.2% suffered depression 6 months after the stroke, considered a score higher than 13 points on the HDS.

Table 1 shows the features and associated factors in the group of patients who scored less than or equal to 13 in HDS compared to those who scored higher than 13. The univariate analysis of the variables shown in Table 1 showed there were statistically significant differences between the studied groups for the following: diagnosis of previous depression (*p* = 0.005), stroke subtype (*p* = 0.020) and laterality (*p* = 0.004), with the severity of the stroke evaluated by the NIHSS at admission (*p* = 0.002), the presence of hemiparesis at discharge (*p* = 0.05), the BI at hospital discharge (*p* = 0.002), the modified Rankin Scale (*p* = 0.001) at discharge and length of hospital stay (*p* <0.001).

For the multivariate analysis (Table 2), a logistic regression model was adjusted for those statistically and/or clinically relevant variables, with the PSD being the dependent variable. The variables introduced in the model were as follows: female gender, previous diagnosis of depression, stroke subtype, location of the stroke, severity of the stroke (score on the NIHS Scale), treatment with intravenous rtPA, hemiparesis at discharge, BI at discharge, mRS at hospital discharge and duration of hospital stay.

After the multivariate analysis, the variables that were independently related to the PSD were previous depression (OR = 2.087 95% CI = 1.01–5.23; *p* = 0.05), the mRS in the time of hospital discharge (OR = 1.296 95% CI = 1.02–1.64; *p* = 0.032) and hospital stay (OR = 1.106 95% CI = 1.01–1.21; *p* = 0.012). The variables described above behaved as independent predictors of PSD.

## 4. Discussion

Physical and psychological well-being is categorically affected after a stroke. In this study, the different variables that could predict the depressive mood of patients after stroke were analyzed. We tried to prove the connections among these variables. Thus, we found the following as predictors: the type and severity of stroke, laterality, functional status at hospital discharge (BI and mRS), hospital stay and previous depression.

The inclusion of these types of evaluations for the early detection of depression (or even with these predictive factors, being able to start treatment early) could have important benefits in the health of the patient and of the health systems themselves; according to some studies, early administration of antidepressants showed advantages in reducing HDS score, improving NIHS scale and improving functional status [38,39].

One meta-analysis of 43 studies reported a cumulative incidence of depression of 39–52% within 5 years following a stroke, results that coincide with the findings of our work (42.2%) [9]. Some studies warn that rates of PSD are higher in the first year after the stroke [7,9,40].

Large and multiple strokes are also predictive of higher frequencies of PSD [41], suggesting that greater neurological tissue loss (sometimes also referred to as stroke severity) serves as a risk factor for PSD. Currently, the evidence revealed that stroke severity could be one of the most important risk factors for PSD, which conformed to the hypothesis that there may be an association between the extent of brain damage and depression. There is evidence that the likelihood of depression increases with stroke severity [42], severity that in our study was assessed by the NIHS scale, which appears as an independent predictor.

Consequently, stroke severity was identified as a vital factor for PSD because of its influence on the levels of handicap and independence that should remain as significant risk factors considered in the prevention of PSD [43]. Movement disorders, dysfunction and life obstacles caused by brain damage could probably lower the self-confidence of patients, and sudden stroke can also be regarded as a negative event for the sufferers, which might increase the incidence of depression.

The association between localized brain lesion after stroke and PSD has been one of the most debated and inconsistent topics of research. This is a controversial aspect for which not all studies have confirmed such a connection. A recent meta-analysis established that the left hemisphere had an association with PSD in the acute and subacute stages [7,43]. Mitchell and colleagues [8] confirmed a statistically significant difference, with PSD occurring in 34% of left-sided lesions, versus 18% of right-sided lesions (OR = 1.50; 95% CI = 1.29–1.74), a result that confirms our findings. However other studies rejected this view and suggest that left-sided strokes are not associated with PSD [27,44,45]. These findings highlight the need to conduct more longitudinal studies assessing anatomical location of the stroke and rates of PSD [13,23].

Some studies analyze the possible reasons that sustain this connection. They conjecture that the left hemisphere is the dominant hemisphere responsible for positive emotions and language, and that the degree of neurological deficits in the left hemisphere is more serious in stroke patients according to the contrast of imaging data [46].

On the other hand, brain damage caused by stroke is located mostly in the frontal lobe and basal ganglia, which change the heart of the emotional network [47] and then lead to depressive symptoms. These findings appear in our work and connect to Chatterjee et al. [48] who indicated that PSD was associated with the focus of stroke, especially the frontal pole, basal ganglia, thalamus and deep white matter lacunar infarction.

At the same time, it is worth noting that using repetitive transcranial magnetic stimulation to stimulate focal brain activity is found more effective when it is administered to the left dorsolateral prefrontal cortex in patients with depression [11]. This phenomenon also suggests that the prefrontal cortex plays an important role in depression.

Regarding hospital stay and the score on the NIHS scale, they could be managed as indirect variables of the severity of the stroke and the sequelae of the stroke. It would be thought that those patients with a greater severity in the stroke and greater functional dependence will have higher scores in the NIHS scale and longer stays in the hospital, in order to stabilize their clinical situation.

PSD is associated with severe disability and functional impairments with regard to activities of daily living (ADL). Those results are connected to our work, where patients that have a higher functional dependence (measured with the mRS and BI) have a greater prevalence of depression, and depression is more serious in PSD [4,15,43,49,50]. The present research suggests a bidirectional relationship between PSD and post-stroke disability [11,51]. In our study, the functional state evaluated by the Barthel Index at discharge and the modified Rankin Scale behave as independent predictors of the multivariate analysis.

Moreover, it has to be taken into account that depression can negatively affect a patient’s ability to actively participate in rehabilitation therapies, falling into a vicious circle, since it has been proven that post-stroke depression was found to have negative effects on functional recovery [4,52,53], including physical exercise, which may provide a complementary treatment for depression. Exercise may affect depressive symptoms through several mechanisms, for example, the hypothalamic–pituitary–adrenal axis may be dysregulated in depression, resulting in elevated cortisol levels [54].

The level of handicap reflects the degree of disability as well as the degree of brain damage.

Regarding depression prior to stroke, some studies [43,50,53] describe patients with a history of depression that were at higher risk of suffering PSD (OR = 2.93, 95% CI = 1.42–6.05). It suggests that a medical history of depression or other psychiatric disorders is one of the leading risk factors for PSD [55]. These results are similar to the findings obtained in our studies (OR = 2.226).

This connection is complicated by the bidirectional relationship between depression and stroke: stroke increases the risk of PSD, but depression is an independent risk factor for stroke and stroke mortality [4,39].

This finding indicates that the patients with pre-stroke depression would have been prescribed with appropriate medication for PSD soon after the onset of stroke [23].

No gender effect was found (*p* = 0.061) as in other studies [56]. However, other articles reported sex (female) was a risk factor for PSD in the acute stage and subacute stage (3 months) [43,53,55].

No significant differences were found in the PSD variable in thrombolyzed patients, such as the Grabowska-Fudala study [57]; although, as they concluded, it is very likely that thrombolyzed patients had more severe neurological deficits in the acute passage. It can be assumed that if thrombolysis had not been used, depressive symptoms would have been more frequent.

Finally, social support was not associated with PSD as was found in the study by Schottke [51]; a common feature of Scohottke’s study with our research is that validated tools were used to assess social support. However, other studies do establish a lack of social support that is associated with a higher risk for PSD [7]. It should be noted that in most studies addressing this topic, social support was operationalized indirectly as a demographic or socioeconomic characteristic, such as the marital or resident status of stroke survivors [58]. It has been hypothesized that social support exerts its protective effects through emotional support, motivation for treatment and support with daily functioning [58].

As to the limitations of the study, it can be mentioned that the rehabilitation carried out has not been evaluated. Some of the questionnaires were answered by proxies, which is the most common method used in assessing patients with aphasia [59]; however, their assessment should be used with caution [60]. The study is limited by its observational design, which may show associations between variables but does not reveal causal and temporal relations. Additionally, we collected a convenience sample with the risk of selection bias.

## 5. Conclusions

PSD is highly prevalent in the follow-up of stroke survivors, and it is associated with the diagnosis of depression prior to stroke, the severity of the stroke, the left location of the stroke, the duration of hospital stay as well as the degree of disability at discharge (BI and mRS).

It seems clear that PSD is a prevalent issue, and its impact on the survivor’s life justifies the evaluation and early treatment that still today continues to be a challenge.

Our findings support the need for a regular assessment and active screening for depressive symptoms in stroke survivors and recognize that depression is not a normal consequence of stroke.

## Figures and Tables

**Table 1:** Sample characteristics. Univariate analysis.

	HRSD ≤ 13 *n* = 100 (57.8%)	HRSD > 13 *n* = 73 (42.2%)	*p*-Value
Mean Age (SD)	70.6 (SD:12.8)	69.7 (SD:11.6)	0.619
Gender Female	30.0%	43.8%	0.061
Gijon´s SFES (SD)	7.74 (SD:1.9)	7.62 (SD:1.8)	0.675
Marital Status			0.363
Married and living together	63.6%	72.6%	
Family	18.2%	16.4%	
Single, separated, or widowed	18.2%	11.0%	
Education			0.243
No studies	8.0%	16.4%	
Primary	73.0%	65.8%	
Secondary	11.0%	13.7%	
Tertiary	8.0%	4.1%	
About STROKE and sequelae			
NIHSS, mean (SD)	5.31(SD: 5.6)	8.22 (SD: 6.7)	0.002
Hemiparesis at discharge	48.0%	63.0%	0.05
rtPA IV	11.0%	20.5%	0.083
Aphasia	27.0%	35.6%	0.072
Stroke Subtype			0.020
TIA	17.0%	17.8%	
PAC	24.0%	8.2%	
TAC	10.0%	28.7%	
POC	20.0%	24.6%	
LAC	18.0%	9.6%	
Hemorrhage	11.0%	10.9%	
Laterality			0.04
Posterior	47.9%	56.7%	
Left	34.4%	38.8%	
Right	17.7%	4.5%	
Functional Status			
Barthel Index discharge (SD)	82.35 (SD: 25.6)	68.29 (SD: 31.8)	0.002
mRS discharge (SD)	1.86 (SD: 1.4)	2.67(SD: 1.6)	0.001
Other variables			
Charlson Comorbidity Index (SD)	1.28 (SD: 1.4)	1.29 (SD: 1.3)	0.971
Previous depression	12.0%	23.3%	0.05
Previous stroke	24.0%	27.4%	0.612
Stay length (days) (SD)	6.10 (SD 3.3)	9.32 (SD6.4)	<0.001

HRSD: Hamilton Depression Rating Scale; TIA: transient ischemic attack; TAC: total anterior circulation stroke; LAC: lacunar stroke; PAC: partial anterior circulation stroke; POC: posterior circulation stroke. NIHSS: National Institutes of Health Stroke Scale; BI: Barthel Index; mRS: modified Rankin Scale. SD: standard deviation.

**Table 2:** Logistic regression analysis for PSD.

Factor	Univariate				Multivariate			
	OR	(IC 95%)		LR-Test; *p*	OR	(IC 95%)		LR-Test; *p*
Gender Female	0.549	0.29	1.03	0.061				
Previous depression	2.226	0.98	5.01	0.051	2.087	1.01	5.23	0.05
Stroke subtype				0.020				
TIA	ref	ref	ref					
PAC	2.375	0.83	6.76					
TAC	5.157	1.62	16.39					
POC	1.221	0.38	3.94					
LAC	1.143	0.35	3.78					
Hemorrhage	3.167	0.79	12.75					
Laterality				0.027				
Right	ref	ref	ref					
Left	4.681	1.28	17.18					
Posterior	4.465	1.18	16.89					
NIHSS, mean (SD)	1.080	1.02	1.14	0.002				
rtPA IV	2.092	0.89	4.87	0.085				
Hemiparesis at discharge	0.846	0.99	3.41	0.049				
Barthel Index at discharge	0.983	0.97	0.99	0.002				
mRS at discharge	1.428	1.16	1.76	0.001	1.296	1.02	1.64	0.032
Stay length (days)	1.169	1.08	1.27	<0.001	1.106	1.01	1.21	0.012

Ref: reference category; OR: odds ratio; CI: confidence interval.
